# PARTNER: A Toolbox for Patient and Public Involvement Governance Within Health Professions Education Research

**DOI:** 10.1111/tct.70477

**Published:** 2026-07-17

**Authors:** Megan Brown, John Norton, Madiha Sajid, Jamie Stogden, Bryan Burford, Gill Vance, Workforce Voices

**Affiliations:** ^1^ School of Medicine Newcastle University Newcastle UK; ^2^ Public Contributor, Workforce Voices Research Partnership

## Abstract

The PARTNER framework offers a practical approach to patient and public involvement governance within health professions education research. It has been created by the NIHR‐funded Workforce Voices Partnership, drawing on the experience of academics, operations staff and public contributors in establishing PPI panels. Although there is agreement that patient and public involvement matters for health professions education research, there is little accessible guidance regarding the governance required to support it. Governance encompasses the structures, decisions and practical arrangements that enable patient and public involvement to be fair, purposeful and sustainable. This includes logistics but also the principles and expectations that help panels function well. Clearer guidance is needed to enable research teams to move from broad commitments to coproduction of research, towards establishing and maintaining conditions that allow for meaningful, sustainable involvement. The PARTNER framework is built around seven areas of governance. Workforce Voices have found key to facilitating meaningful involvement: (1) payment and recognition; (2) agreement; (3) roles and responsibilities; (4) training and support; (5) networking; (6) equity of recruitment and membership; and (7) review, evaluation and feedback. Each area of the framework is linked to practical tools, templates and tips that can be adapted for readers' own research projects. The aim of this toolbox is to offer a structure for novice or developing researchers to navigate PPI governance with confidence and in a way that promotes meaningful engagement and involvement.

## 1 Introduction

Whereas patient and public involvement (PPI) is well established within Health Professions Education (HPE) [1, 2, 3, 4], calls for greater focus on PPI within HPE *research* are more recent [5]. Funders and journals increasingly expect meaningful PPI [6, 7], recognising its role in enhancing relevance, accountability and equity [8, 9].

In HPE, ‘PPI’ also includes the participation of clinical staff, educators, students and trainees [10], reflecting their dual roles as professionals and stakeholders in educational systems. Whereas the value of their involvement is increasingly well articulated [11], the processes that underpin its effective governance remain far less well‐described.

Governance refers to the process (and associated activities) of establishing and overseeing the management of PPI within HPE studies. Weak or absent governance can limit PPI impact and negatively affect inclusivity [12]. For PPI to be successful and sustainable, attention to the governance required to support work is critical [7].

We are a team of patient and public representatives, researchers and operations staff working within the NIHR‐funded Workforce Research Partnership ‘Workforce Voices’. Workforce Voices addresses recruitment, retention and workforce sustainability in underserved areas of the NHS [13]. We have established two PPI panels, one for staff and one for patients/public. Separate panels support psychological safety, and there are opportunities for groups to communicate via wider project governance, for example, monthly management groups, though this is a design choice rather than a universally applicable model to panel construction. Both patients and staff have important perspectives on workforce sustainability—staff from front‐line clinical experiences, patients from experiences of accessing care and care continuity/quality.

Although existing literature emphasises working in partnership [9, 12], it provides little detail on operational frameworks to enact this, especially within HPE, and accessible governance resources for establishing PPI panels remain scarce. In response, we developed the PARTNER framework, which sets out seven governance building blocks that we developed to support transparent, equitable and sustainable PPI practice. By sharing our processes and example documents (which may be used as templates), we aim to support others in developing robust PPI governance for HPE research.

*We developed the PARTNER framework, which sets out seven governance building blocks that we developed to support transparent, equitable and sustainable PPI practice*.

This work has been enabled by NIHR Health and Social Care Delivery Research funding, which supported operational roles and payment for contributors. Not all research teams have access to comparable resources, but meaningful involvement is still possible, for example, through flexible involvement and working with community groups. Throughout, we will indicate options for researchers working in different resource settings. We also hope our templates reduce the cost of engaging with PPI governance.

## 2 PARTNER: The 7 Building Blocks of PPI Panel Governance

We developed the PARTNER framework by reflecting on the early stages of convening and supporting our panels, paying close attention to where key governance questions arose as we translated our ethos of coproduction into PPI practice. Each building block is associated with practical tools.

*Each building block is associated with practical tools*.

**PARTNER** = **P**ayment and recognition; **A**greement; **R**oles and responsibilities; **T**raining and support; **N**etworking; **E**quity of recruitment and membership; **R**eview, evaluation and feedback.

### 2.1 Payment and Recognition

Fair payment for time and expertise is key to equitable PPI. NIHR guidance (2024) highlights remuneration as a way of recognising lived experience as expertise. When involvement relies on goodwill alone, exclusion becomes more likely [14]. Costing PPI within funding bids, or drawing on institutional budgets/small grants, helps ensure necessary resources are available.

In Workforce Voices, payment rates were informed by NIHR guidance on public involvement and aligned with expected time commitment. Our £55/meeting rate reflects 2 h of involvement, including preparation time, consistent with national guidance at project outset. Researchers may use NIHR guidance as a benchmark but should also consider their local context—for example, if working with local community organisations, groups may have their own payment rates. Rates that exceed those used by local groups can create tension, whereas lower rates can limit inclusion. Early discussion with community partners can help establish payment rates.

Providing clear information about payment routes, timelines and required documentation builds trust and supports informed decision‐making. Operational and administrative support is invaluable for navigating university finance systems—we recommend costing this into funding bids. Where this is not possible, advocate for support within your institution using the logic that support is key to meeting funder expectations, creating impactful research and financial accountability. An upfront investment can save time and money down the line.

Researchers who cannot access support can develop their knowledge of payment by meeting with their finance departments or organisational PPI leads. We have found other research groups within our institutions open to sharing their processes, which enabled us to develop our understanding of payment. Begin as soon as possible, as understanding financial processes can take time.

Recognition should extend beyond payment [8] and, in resource constrained settings, may take other forms, with transparent recognition of funding limitations to PPI members. Contributors should be offered authorship on outputs where appropriate, in line with International Committee of Medical Journal Editors (ICMJE) guidance [15], and included in team communications (e.g., update emails, newsletters). Other options include opportunities to copresent work at academic/community events, certificates and online visibility—for example, sharing success via social media. Preferred recognition should be discussed and agreed with panels at outset.

### 2.2 Agreement

Clear agreements between panel members and the research team help establish a shared understanding of a project's purpose, remit and scope [7]. For Workforce Voices, researchers, working with two PPI coapplicants, created a draft Terms of Reference (ToR) to support recruitment and outline objectives, expectations and meeting arrangements (see Table 1).

**TABLE 1:** Example summarised terms of reference, using workforce voices patient/public panel as an example.

Section	Description
Purpose of the panels	The PPI Panels support Workforce Voices by providing insights, feedback and advice so the research reflects diverse perspectives and meets the needs of healthcare staff and the wider public. Panels act as a critical friend, contributing to study design, implementation, and dissemination.
Roles & responsibilities	Advisory: Share ideas to improve working conditions so jobs are more supportive, manageable and rewarding; promote fair and equal opportunities for all careers; and help strengthen project design, communication and activities. Collaboration: Work with the project team to focus on practical solutions and develop clear, useful recommendations from findings. Representation: Bring personal experience, professional knowledge and/or community insights to discussions on workforce and wellbeing. Engagement: Attend meetings; review materials (leaflets, slides, posters and reports); and keep discussions confidential and respectful.
Membership & expectations	Who can join: Healthcare staff, patients, carers and members of the public interested in improving healthcare jobs and services. The Partnership research team may join PPI meetings when required. Tech requirements: Access to a computer/smartphone, reliable internet and an email address.
Meetings	Two panels (staff; patient/public), of 10 members per panel. Meet at least four times per year: Three online and one in person (typically at Newcastle University). A hybrid option is always available. Where in‐person attendance is possible, travel and accommodation costs will be reimbursed; caring costs can be reimbursed case‐by‐case.
Administration & representation	Meetings are supported and minuted by a project team member. Notes and actions are agreed by all. Panel Chairs: Public panel—Madiha Sajid; staff panel—John Norton. Each Chair represents their panel at monthly Partnership Management Meetings.
Time commitment	Meetings last 1 h. Prereading is circulated at least 2 weeks in advance and should take no more than 1 h. Additional workshops/discussions may be offered; these are voluntary and time commitments will be stated in advance. Members may also be asked to share ideas/feedback via email between meetings.
Code of conduct	Create a safe, respectful space. Listen to others; offer constructive feedback. Keep information confidential. Consider ideas on their merits without bias or prejudice.
Payment and renumeration	Reimbursement: £55 per meeting attended (covers ~2 h: 1 h meeting +1 h preparation, plus internet/printing costs). Additional expenses (e.g., caring costs; travel and accommodation for in‐person meetings) may be approved in advance—email the Community Inclusion & Engagement Lead Megan Brown: megan.brown@newcastle.ac.uk; copying the relevant Chair (Madiha Sajid: m.sajid@imperial.ac.uk John Norton: john.norton@uhs.nhs.net).
Accessibility	Materials provided in accessible formats (plain language, large print; additional formats as needed). Support for technology and reasonable adjustments is available—please let us know your requirements.
Training & development resources	Guidance and resources will be provided as necessary to support participation, including explanations of key terms and project goals.
How we use your input	Member input helps shape study design, implementation, and recommendations. We share updates on how feedback is used and acknowledge contributions in all reports and publications. We also seek member feedback on the panels to support continual improvement.

Once panels were established, we codeveloped a shared statement of values and ways of working with members during initial meetings. We brought a draft ToR for discussion and invited members to shape language, expectations and priorities. This included agreeing principles for communication and shared working. Through this process, the ToR shifted from a fixed, procedural document into a living statement which reflects each group's priorities. Ensuring materials are accessible, reviewing them regularly and discussing how they are used has supported trust and mutual accountability [16].

### 2.3 Roles and Responsibilities

Clarity about who does what within PPI is essential for effective collaboration. Ambiguity can lead to confusion and disengagement [17]. At the outset, we outlined the roles of panel members, panel chairs, researchers and operational staff to create a shared understanding of responsibilities and decision‐making processes. Aligning panel activities with SMART (Specific, Measurable, Achievable, Relevant, Time Bound) goals helped connect discussions to wider project aims.

We found it important to be explicit that PPI input, although highly influential, is advisory rather than determinative. Where contributors' views differ, the research team weighs these against project aims, methodological requirements and feasibility. Final decisions sit with the research team but are communicated to contributors transparently, including where suggestions cannot be taken forward and why.

Within Workforce Voices, each panel is cochaired by a public contributor and an academic, supporting shared ownership [18]. Dedicated operational support (administration, finance and project‐wide communication) has been vital yet remains underdescribed in PPI literature [19].

We also developed consent processes for contributors, outlining the purpose of their role, expected activities, likely time commitment, reimbursement arrangements and how contributions may be used. Making this information explicit helps contributors understand what participation involves in practice. It also supports recruitment by enabling early discussion about whether a role is a good fit for a contributor's interests, experience and availability. An example consent form from Workforce Voices is provided (Table 2).

**TABLE 2:** Consent form for public partner project involvement, example from Workforce Voices.

Agreement ID No.
Full name of public partner:
Email address:
Contact telephone number:
Name of project: Workforce voices
Description of project: Workforce Voices is a five‐year project funded by the NIHR. It focuses on improving staffing in primary care and maternity services, especially in areas where support is most needed. The project aims to tackle issues like staff shortages, keeping workers in their roles, and reducing stress and workload. To achieve this, we will work closely with healthcare staff, patients, and the public to design, test, and evaluate new ideas and solutions together.
Description of public partner role and any outputs/deliverables expected: Public partners will: Provide insights and feedback on project activities and outputs. Participate in meetings and contribute to discussions on workforce sustainability challenges and interventions. Advise on the feasibility and acceptability of interventions. Represent patient/public perspectives in the governance of the project.
Expected time commitment and any dates public partners need to be available: Public partners are expected to Attend quarterly meetings (virtual or in‐person) and provide input through occasional surveys or feedback sessions. Participate in key milestone reviews and project codesign workshops as required. Key dates: To be communicated with sufficient notice.
How public partners will be reimbursed: Public partners will be reimbursed at an agreed rate for their time. Travel and subsistence costs will also be covered, in line with the project's payment policy.
Your designated point of contact is: Megan Brown, Community Involvement and Engagement Lead, email address megan.brown@newcastle.ac.uk .
Public partner's consent to participate: I have read, understood and consent to the involvement role I am being asked to play as part of the project/initiative described above, and agree to the terms of reimbursement Signature: ________________________ Date: __________________________.
Public Partners Acceptance of responsibilities in relation to potential income tax liability, entitlement to state benefits and health insurance payments: Please be aware that acceptance of payment in the form of cash, or cash equivalents (i.e., gift vouchers) for your time spent on involvement activities may have implications for your income tax liability and/or entitlement to state benefits and/or receipt of health insurance payments while on sick leave Please refer to *the Workforce Voices Payment Policy for Patients and the Public (Public Partners)* to understand if relevant to you and what action to take. If you have any questions, please speak with your designated contact. I have read and understood the *Partnership for Workforce Sustainability Payment Policy for Patients and the Public (Public Partners)* describing my responsibilities where applicable regarding state benefit, health insurance, and income tax liability. Signature: ________________________ Date: __________________________.
Consent to be contacted about other Involvement opportunities: Please tick the following box if you are happy for Workforce Voices to retain your contact details and contact you about other involvement opportunities should they arise. We will not give your contact details to anyone else without asking you first.

### 2.4 Training and Development Support

Effective PPI requires structured, ongoing support for contributors and researchers. Clear onboarding materials and 1:1/group orientation sessions can help new contributors understand project aims, context and expectations, supporting confidence and belonging [6].

PARTNER understands training as a two‐way process. Although public contributors may have training needs, for example, understanding research methods, researchers often also have training needs relating to designing activities for panels, communicating ideas accessibly and inclusive facilitation [8].

To identify and address needs, we developed a training needs assessment (Table 3), which can be completed by researchers and contributors at outset. This helps tailor support to individual and group experience levels and is revisited annually. Training can be delivered in‐house, by project team members/PPI leads, but it is wise to cost a training budget into funding bids to facilitate external provision as necessary.

**TABLE 3:** Training Needs Assessment, example from Workforce Voices to be shared as an electronic form.

Section	Content
Introduction	This tool aims to identify the skills, knowledge, and support required by panel members to contribute effectively to Workforce Voices. Please complete the sections below to help us tailor training and resources to meet your needs.
1. Personal details (optional)	Name: Email: Preferred contact method: ☐ Email ☐ Phone call ☐ Text
2. Current skills and knowledge	Confidence rating: Please rate your confidence in the following areas on a scale of 1 (*not confident*) to 5 (*very confident*): Understanding the purpose of the PPI panel Participating in group discussions Reviewing and providing feedback on written materials (e.g., reports, posters) Using online meeting tools (e.g., Zoom, Teams) Understanding healthcare workforce challenges Understanding research processes Skills and experience: Please tick all that apply: ☐ Lived experience ☐ Professional background ☐ Community engagement ☐ Research involvement ☐ Healthcare experience (personal or professional) ☐ Advocacy or campaigning ☐ Education or training experience ☐ Leadership or management skills ☐ Public or patient involvement (PPI) experience ☐ Policy or governance experience ☐ Communication or facilitation skills ☐ Analytical or problem‐solving skills ☐ Project management experience ☐ Creative or design skills ☐ Other (please specify): __________
3. Training needs	Areas where you would like more training or support (tick all that apply): ☐ Understanding the role and responsibilities of the PPI panel ☐ Communication skills—verbal (active listening, contributing to discussions) ☐ Presentation skills—delivering ideas clearly to groups ☐ Written communication—summarising discussions, drafting documents ☐ Facilitation skills—guiding group conversations, managing meetings ☐ Providing feedback constructively ☐ Using technology for virtual meetings ☐ Understanding healthcare research ☐ Understanding healthcare workforce issues ☐ Other (please specify): __________ Support required (tick all that apply): ☐ Accessibility adjustments (e.g., large print, screen readers) ☐ Language or translation support ☐ Digital skills training (e.g., how to use Zoom, Teams) ☐ Understanding research terminology ☐ Barriers at home or work that might make taking part in training difficult (e.g., working hours, caring roles, etc.) ☐ Other (please specify): __________
4. Preferred learning methods	Preferred learning formats (tick all that apply): ☐ Online workshops or webinars ☐ In‐person training sessions ☐ Written guides or handouts ☐ Videos or recorded sessions ☐ Other (please specify): __________ Preferred time of day for ‘live’ sessions (tick all that apply): ☐ Morning ☐ Afternoon ☐ Evening
5. Additional comments	Do you have any other comments, suggestions, or concerns about your training needs? [Open text box for response]

All panel materials (including training materials) should be produced in accessible language. Glossaries of common jargon can be useful, as can buddying systems, where new contributors are paired with more experienced panel members.

### 2.5 Networking

Sustained communication (as an internal project network) is central to keeping PPI panels informed and engaged between meetings.

Within Workforce Voices, we have multiple communication mechanisms, using a blend of digital tools (e.g., Teams, shared folders, email newsletters and blog) and in‐person contact to enable flexible participation and support accessibility. These tools facilitate coordination and help maintain a sense of shared purpose by connecting contributors to the wider project.

Regular, proportionate communication (balancing ‘enough’ information to stay connected without overwhelming participants) is key to maintaining engagement. If you are uncertain regarding the ideal frequency of updates, you can ask panels to advise on their preferences. Clear communication also helps avoid the isolation or ‘siloing’ of panels from wider project governance [7].

We share responsibility for engagement with panels equitably across Workforce Voices. Our staff PPI panel, for example, prefer to meet outside standard office hours—we plan for this collectively, so the responsibility does not fall on one person.

Sustaining engagement over time requires adopting a relational approach. In Workforce Voices, this includes regularly providing feedback on how contributors' input has shaped work, offering flexible opportunities for involvement and revisiting preferences regularly. Maintaining a sense of purpose, recognition and responsiveness has been central to supporting engagement.

Although Workforce Voices is ongoing, we have begun to consider how relationships with contributors will be brought to a close considerately. Planning for the end of a project at the project outset might seem strange, but it facilitates clear communication about timelines and supports opportunities for continued involvement where appropriate (e.g., dissemination). Where formal involvement ends, relationships can be sustained through light‐touch communication (e.g., sharing outputs/impacts), development of an alumni‐style network and signposting to future opportunities. This helps avoid abrupt endings, while managing expectations regarding limits of unfunded engagement. Supporting Information S2 provides a checklist of key considerations relating to engagement.

## 3 Equity of Recruitment and Membership

Supporting diversity within PPI panels requires intentional, sustained effort. Recruitment needs to extend beyond an open advert to include targeted outreach that reaches communities often underrepresented in research. Barriers to involvement (e.g., digital exclusion, time pressures and language barriers) are common [20] and should be addressed practically, including via reimbursement of caring costs, translation and accessible materials [8, 21]. Where recruitment materials are translated, support should extend into meetings (e.g., via live interpretation, captioning and provision of translated materials). Costs will depend on project scale/size, but budgeting requires careful planning and is a sizeable but necessary cost. We recommend working with experienced PPI leads, community organisations or institutional groups (e.g., PPI groups within the UK NIHR Research Support Service) to develop costings.

It is sensible to create a recruitment strategy prior to approaching PPI networks or groups. Workforce Voices' recruitment strategy is offered as an example (Table 4). We understand recruitment of contributors to be ongoing, rather than a one‐off process. Panel membership is reviewed annually, or sooner if needed, to support the capacity of contributors and representation across panels.

**TABLE 4:** Recruitment strategy, Workforce Voices.

Section	Description
Objective	To establish diverse and representative Patient and Public Involvement (PPI) and Professional Panels that contribute effectively to the Partnership's goals of workforce sustainability, equity, and inclusion.
Key activities	Leverage existing networks: Use expressions of interest gathered during earlier PPI activities (reach out to Healthwatch, Newcastle Health Research Partners, NIHR Research Support Service). Engage with Voice Global to create recruitment materials and advert for their platform. Develop and advertise recruitment materials: Produce accessible advertisements, including lay summaries and easy‐read, pictorial versions. Address accessibility needs (translations, alternative formats) identified during recruitment. Targeted outreach: Disseminate adverts through regional and national networks, including: Professional networks (e.g., Royal College of General Practitioners, Deep End General Practice Networks, Royal College of Obstetrics and Gynaecology). Social media (hashtags: #PPI, #PatientInvolvement, #HealthResearch, #MedicalResearch, #PublicHealth)—Bluesky, X, LinkedIn Online communities (e.g., Mumsnet; Facebook groups such as Birth Trauma Association Parent Support Group, National Maternity Voices, Bliss, Gateshead Maternity and Neonatal Voices Partnership, Resilient GP, GPUK, Chronic Illness communities). Note: Many professional groups are closed; posts to be made by members where possible/with consent of group admins, accessible by direct message. Community locations (pharmacies, churches, libraries). Explore local engagement routes with Healthwatch. JN to lead on grassroots connections.
Application & screening process	Applicants invited to complete a short written application outlining their interest in the panel, relevant experiences or perspectives, and any support or adjustments required to participate. Interested individuals are asked to provide written responses to three questions: Why are you interested in joining the panel? What skills, experiences, or perspectives do you bring? How do you think your involvement could make a difference? Applications reviewed using a structured scoring approach based on each question asked. The questions were developed iteratively by the research team, drawing on NIHR guidance for inclusive involvement and refined through discussion to ensure alignment with the aims of the Partnership and the values underpinning the panels. Scoring is undertaken by more than one member of the research team (e.g., PPI academic lead, and a PPI coapplicant) to support consistency and reduce individual bias. Please see Supplementary Material for the associated scoring rubric. The aim is not to select the most ‘qualified’ applicants, but to build a panel with a complementary range of experiences and perspectives. All scorers should meet to discuss the scoring of their applications, which will inform who to invite to interview (the top 15 scoring members per panel). Before the meeting, one individual should collate the dual scores, and calculate ranking based on score combination. All applications should then be discussed in the meeting, recommend 2–3 h meeting to facilitate discussion regarding selection. Short informal interviews are then conducted with the top scoring members to explore their interests and perspectives further and to ensure that applicants have a clear understanding of the role/have the opportunity to ask questions. Informal interviews held by panel chairs, who are PPI coapplicants. Interviews are not scored formally, but the interviewer makes notes after each short interview, reflecting on the areas assessed by the rubric. At the end of all interviews, the interviewer makes a recommendation to the team regarding appointing to their panel. Diversity monitoring questions, informed by NIHR guidance are sent to all panel members via an online form, and completed on a voluntary basis, to avoid creating barriers at the point of application and to support appropriate data governance. All applicants receive feedback following the process by email. If there are a large quantity of written emails, the email informing them they have not been successful may be generic, but should be populated with broad observations of strengths and areas for improvement across all applications. All who are interviewed should receive written feedback by email, or verbal feedback by telephone call or Teams—whatever is most accessible to them/preferred by them. This should be framed to acknowledge the strengths of their application, clarify the outcome, and where appropriate, suggest other opportunities for involvement. Providing feedback is important to maintain trust and to avoid reinforcing exclusion for those not selected.
Notification & confirmation	Notify selected candidates, confirm participation, and provide next steps. Maintain communication if delays occur between selection and start. Create a reserve list of appointable candidates for future vacancies. Provide supportive feedback to all applicants and invite continued engagement with Partnership activities.
Panel composition criteria	Patient/public panel (total *n* = 10): Representation from underserved groups (e.g., rural/coastal areas, socioeconomic deprivation). Inclusion of individuals with lived or caring experience related to health inequalities, long‐term conditions, or primary/maternity care (≥ 6 months). At least one‐third of members to be new to PPI (≤ 6 months of experience). Initial round of recruitment: 3 members—1 with primary care experience, 1 with maternity care experience, 1 new to PPI. Professional Panel (total *n* = 10): Clinical and nonclinical professionals from primary care, social care, and maternity services across diverse regions and protected characteristics (as defined by the Equality Act 2010, including age, disability, gender reassignment, marriage and civil partnership, pregnancy and maternity, race, religion or belief, sex and sexual orientation). Initial round of recruitment: 3 members—1 primary care professional, 1 maternity care professional and 1 professional from the nursing/midwifery/allied health professions (NMAHP+).
Accessibility & inclusion measures	Reimbursement of expenses, childcare or caring costs; translation and accessible document formats; flexible meeting scheduling; hybrid participation options.
Review & refresh	Recruitment should be viewed as ongoing, not one‐off. Membership reviewed annually (or sooner if needed) to maintain diversity, energy, and relevance.

Supporting equity also means being politically and socially aware. This means acknowledging the wider context of inequality and discrimination that may affect contributors' confidence to participate and attending to intersectionality (e.g., how disability and ethnicity interact to shape access/participation). In practice, this includes offering multiple ways to contribute (e.g., verbal, written and asynchronous); reviewing who attends, and contributes within, meetings; working with community organisations to reach underrepresented groups; and revisiting support needs regularly. Creating space for (voluntary) dialogue about these dynamics and offering support can also facilitate inclusion.

### 3.1 Review, Evaluation and Feedback

Evaluating PPI activity is an important way we can evidence impact, sustain engagement and improve practice over time [22].

Within Workforce Voices, evaluation is embedded and ongoing rather than one‐off. We use several complementary methods—including impact registers (Table 5 shows our example template, which we codevelop with panels, and maintain in a shared Excel file) and annual surveys—to capture how PPI contributions influence project design, outputs and dissemination. Feedback loops are critical—panel members are shown how their input shaped decisions and outcomes to maintain motivation and trust. However, we recognise the risk of ‘evaluation fatigue’ among contributors—panels should be consulted on preferred intervals and methods of feedback/evaluation.

**TABLE 5:** Example impact register template from Workforce Voices.

Category	Description/prompts	Example entry
Date/activity	What activity, meeting or event did this feedback relate to?	Staff panel meeting—April 2025
Panel/group	Which PPI or professional panel provided input?	Patient and public panel
Topic/focus area	What aspect of the project did the feedback concern?	Accessibility of survey materials
Contributor input/feedback summary	What key points, ideas or concerns were raised?	Suggested using plain language and including examples of rural practice contexts
Research team response/action taken	What actions or changes were made as a result?	Survey revised and pilot‐tested with two panel members
Stage of project influenced	Which phase of the project was affected (e.g., design, analysis and dissemination)?	Design
Impact type	Nature of impact: conceptual (understanding), instrumental (practical change) or relational (collaboration/trust).	Instrumental
Outcomes/evidence of change	What was the result or visible outcome of this input?	Revised survey improved clarity and participant response rate
Feedback to contributors	How and when were contributors informed of the impact of their input?	Summary shared in next newsletter and meeting update
Reflections/learning	What was learned about the process, facilitation or partnership?	Need to involve panel earlier in drafting stage
Next steps/follow‐up	What actions or monitoring are planned?	Schedule early draft review for next data collection phase

## 4 Lessons Learned

As Workforce Voices is still within its first year, these lessons are based on our experience of early implementation of PARTNER rather than formal evaluation.

Establishing effective governance for panels has relied on clear structures, trust and communication. Dedicated operational support (JS) has been central, particularly for navigating university systems and finance processes. This role needs to be costed into bids and appointed early. Having a clear academic lead for PPI (MB) has provided strategic direction across Workforce Voices.

Building accessibility into every stage of panel design has been key—positive impacts are evident via a strong recruitment response and positive informal feedback from members. Investing time in relationship‐building during onboarding, supported by a light‐touch application and interview process (Table 4), helped foster trust prior to meetings.

Connecting PPI structures with wider project governance has strengthened the visibility and influence of panel contributions. Panel chairs (JN and MS) attend Partnership Management Group and Research Advisory Group meetings, ensuring perspectives are carried through decision‐making.

There have been some difficulties, however. Before operational support was in place, payment processes were slow and confusing. Early panel meetings were dominated by codeveloping governance, and it was important to manage expectations about the importance of taking this time. Recruitment remains an area for reflection, as early cohorts are more homogeneous demographically than we had hoped. This may reflect limitations of relying on existing PPI networks for recruitment, alongside structural barriers (e.g., time and digital access). In our annual panel reviews, we plan to consider which perspectives are underrepresented and work with community organisations to diversify recruitment.

As stated within our introduction, we appreciate that PARTNER was developed within a UK NIHR‐funded programme of research, with established infrastructure for PPI and dedicated operational support. Implementation in other contexts is likely to require adaptation as previous research shows involvement practices are shaped by local organisational structures and resource availability [7, 12]. In settings with fewer resources, governance activities may need to be integrated into existing community structures or supported through partnerships with local organisations. Similarly, approaches to payment, communication and panel membership may require modification to align with local priorities or cultural expectations. We recommend readers see PARTNER as an adaptable framework whose principles can be operationalised flexibly, depending on context and potential resource constraints, rather than a prescriptive model. Future PPI scholarship utilising PARTNER, and reflective outputs regarding use of the framework, would help identify which principles of the framework require contextual refinement depending on setting.

*In settings with fewer resources, governance activities may need to be integrated into existing community structures or supported through partnerships with local organisations*.

## 5 Conclusion

Governance tools cannot remove the complexity of convening and managing PPI panels, but they support teams to navigate complexity with greater clarity and confidence. Coordinating PPI is often invisible work, yet it is foundational to its success within HPE research. The PARTNER framework makes clear key considerations for building PPI structures that are fair, transparent and sustainable. By making governance visible and sharing openly our templates, we aim to support HPE research teams to create inclusive and supportive environments for PPI.

*Governance tools cannot remove the complexity of convening and managing PPI panels, but they support teams to navigate complexity with greater clarity and confidence*.

## Author Contributions

**John Norton:** resources, writing – review and editing, funding acquisition, project administration. **Megan Brown:** conceptualization, writing – original draft, writing – review and editing, funding acquisition, project administration. **Madiha Sajid:** resources, conceptualization, funding acquisition, writing – review and editing, project administration. **Jamie Stogden:** resources, writing – review and editing, conceptualization, project administration. **Bryan Burford:** supervision, writing – review and editing, conceptualization, funding acquisition, project administration. **Gill Vance:** funding acquisition, conceptualization, writing – review and editing, project administration, supervision.

## Funding

This work was supported by the National Institute for Health and Care Research (NIHR160772).

## Conflicts of Interest

The authors declare no conflicts of interest.

## Supporting information


**Data S1:** Scoring rubric for PPI panel applications.
**Data S2:** Checklist of considerations for sustaining PPI panel engagement.

## Data Availability

Data sharing is not applicable to this article, as no datasets were generated or analysed during the current study.

## References

[1] R. L. Baines and S. Regan de Bere , “Optimizing Patient and Public Involvement (PPI): Identifying Its “Essential” and “Desirable” Principles Using a Systematic Review and Modified Delphi Methodology,” Health Expectations 21, no. 1 (2018): 327–335. 28929554 10.1111/hex.12618 PMC5750770

[2] A. Jawwad , A. Sajid , S. Ramani , H. Popeijus , and M. Govaerts , “Active Patient Involvement in Healthcare Education in the Global South: Perspectives From Pakistan,” Clinical Teacher 22, no. 5 (2025): e70184. 40858519 10.1111/tct.70184 PMC12380654

[3] S. Regan de Bere and S. Nunn , “Towards a Pedagogy for Patient and Public Involvement in Medical Education,” Medical Education 50, no. 1 (2016): 79–92. 26695468 10.1111/medu.12880

[4] P. Rowland , K. R. MacKinnon , and N. McNaughton , “Patient Involvement in Medical Education: To What Problem Is Engagement the Solution?,” Medical Education 55, no. 1 (2021): 37–44. 32350875 10.1111/medu.14200

[5] B. Burford , P. Yeates , A. H. Bluemel , et al., “Establishing Priorities for Clinical Education Research: Exploring the Views of UK Professional and Public Stakeholders,” Clinical Teacher 22, no. 4 (2025): e70144. 40588837 10.1111/tct.70144 PMC12209556

[6] M. S. Y. Biddle , A. Gibson , and D. Evans , “Attitudes and Approaches to Patient and Public Involvement Across Europe: A Systematic Review,” Health Expectations 24, no. 3 (2021): 802–813. 10.1111/hsc.13111 32705752

[7] T. Greenhalgh , L. Hinton , T. Finlay , et al., “Frameworks for Supporting Patient and Public Involvement in Research: Systematic Review and Co‐Design Pilot,” Health Expectations 22, no. 4 (2019): 785–801. 31012259 10.1111/hex.12888 PMC6737756

[8] J. O. Brett , S. Staniszewska , C. Mockford , et al., “Mapping the Impact of Patient and Public Involvement on Health and Social Care Research: A Systematic Review,” Health Expectations 17, no. 5 (2014): 637–650. 22809132 10.1111/j.1369-7625.2012.00795.x PMC5060910

[9] P. Wilson , E. Mathie , J. Keenan , et al., “Research With Patient and Public Involvement: A Realist Evaluation—The RAPPORT Study,” Health Services and Delivery Research 3, no. 38 (2015): 1–176. 26378332

[10] É. Ambrosetti , C. Gaudin , S. Flandin , and G. Poizat , “Students as Co‐Designers in Health Professional Education: A Scoping Review,” BMC Medical Education 25, no. 1 (2025): 645. 40319259 10.1186/s12909-025-07110-0 PMC12049780

[11] K. D. Könings , S. Mordang , F. Smeenk , L. Stassen , and S. Ramani , “Learner Involvement in the Co‐Creation of Teaching and Learning: AMEE Guide No. 138,” Medical Teacher 43, no. 8 (2021): 924–936. 33153367 10.1080/0142159X.2020.1838464

[12] J. Ocloo , S. Garfield , B. D. Franklin , and S. Dawson , “Exploring the Theory, Barriers and Enablers for Patient and Public Involvement Across Health, Social Care and Patient Safety: A Systematic Review of Reviews,” Health Research Policy and Systems 19, no. 1 (2021): 8. 33472647 10.1186/s12961-020-00644-3 PMC7816359

[13] M. E. Brown , E. Hoverd , A. Montgomery , B. Burford , and G. Vance , “When I Say … Workforce Sustainability,” Medical Education 60, no. 5 (2025): 487–489. 41065583 10.1111/medu.70072 PMC13067067

[14] M. Madden , and E. Speed , “Beware Zombies and Unicorns: Toward Critical Patient and Public Involvement in Health Research in a Neoliberal Context,” Frontiers in Sociology 2 (2017): 262366.

[15] International Committee of Medical Journal Editors (ICMJE) , “Recommendations for the Conduct, Reporting, Editing, and Publication of Scholarly Work in Medical Journals,” (2026), http://www.icmje.org/recommendations/. 10.12771/emj.2024.e48 PMC12093628 40704003

[16] J. Ocloo , and R. Matthews , “From Tokenism to Empowerment: Progressing Patient and Public Involvement in Healthcare Improvement,” BMJ Quality & Safety 25, no. 8 (2016): 626–632. 10.1136/bmjqs-2015-004839 PMC4975844 26993640

[17] N. D. Shippee , J. P. Domeq Garces , G. J. Prutsky Lopez , et al., “Patient and Service User Engagement in Research: A Systematic Review and Synthesized Framework,” Health Expectations 18, no. 5 (2015): 1151–1166. 23731468 10.1111/hex.12090 PMC5060820

[18] C. Mockford , S. Staniszewska , F. Griffiths , and S. Herron‐Marx , “The Impact of Patient and Public Involvement on UK NHS Health Care: A Systematic Review,” International Journal for Quality in Health Care 24, no. 1 (2012): 28–38. 22109631 10.1093/intqhc/mzr066

[19] L. Louise and B. Annette , “Drawing Straight Lines Along Blurred Boundaries: Qualitative Research, Patient and Public Involvement in Medical Research, Co‐Production and Co‐Design,” Evidence & Policy 15, no. 3 (2019): 409–421.

[20] S. Hatch , J. Fitzgibbon , A. J. Tonks , and L. Forty , “Diversity in Patient and Public Involvement in Healthcare Research and Education—Realising the Potential,” Health Expectations 27, no. 1 (2024): e13896. 37867364 10.1111/hex.13896 PMC10726264

[21] National Institute for Health and Care Research (NIHR) , “Working With People and Communities,” (NIHR, 2024), https://www.nihr.ac.uk/research‐funding/application‐support/working‐with‐people‐and‐communities.

[22] S. Staniszewska , A. Adebajo , R. Barber , et al., “Developing the Evidence Base of Patient and Public Involvement in Health and Social Care Research: The Case for Measuring Impact,” International Journal of Consumer Studies 35, no. 6 (2011): 628–632.

