# Supplementary material for: PARTNER: A Toolbox for Patient and Public Involvement Governance Within Health Professions Education Research
Source: Clin Teach. 2026 Jul 17;23(4):e70477. doi: 10.1111/tct.70477 (PMC13377659; doi:10.1111/tct.70477)
Supplement: Supplementary file 1 — Data S1: Scoring rubric for PPI panel applications. Data S2: Checklist of considerations for sustaining PPI panel engagement. [file TCT-23-e70477-s001.docx]

## Supplementary material 1

**Scoring rubric for PPI panel applications**

**Scoring scale:**
0 = Not addressed
1 = Some evidence

2 = Strong evidence

| **Criteria** | **Description** | **Assessed using the following written question** | **Score (0–2)** |
| --- | --- | --- | --- |
| Motivation | Clarity of interest in the panel and broader project | Why are you interested in joining the panel? | 0–2 |
| Lived experience or perspective | Relevance and depth of experience or insight offered | What skills, experiences, or perspectives do you bring? | 0–2 |
| Understanding | Understanding of the panel’s purpose, the broader programme of work within which the panel sits/issues relevant to the programme of work | How do you think your involvement could make a difference? | 0–2 |

**Total possible score: 6**

## Supplementary material 2

**Checklist of considerations for sustaining PPI panel engagement**

The purpose of this short checklist is to support research teams in maintaining meaningful relationships with PPI contributors over time, and ending involvement in a transparent and considerate way. It is structured by stage of project.

**At project outset**

☐ Have timelines for PPI involvement throughout the project been clearly communicated?

☐ Have you discussed how contributors’ input will be used throughout the course of the project?

☐ Have you considered how relationships will be sustained and concluded?

☐ Have expectations about ongoing involvement beyond funding been made explicit?

**During the project**

☐ Are contributors receiving regular, proportionate updates?

☐ Are multiple modes of participation available (e.g. meetings, written, asynchronous)?

☐ Are you reviewing who is able to attend, and contribute within, meetings?

☐ Are support needs revisited over time (not assumed to be fixed)?

☐ Are contributors updated on how their input is shaping the work? (You may wish to consider creating an Impact Register)

**Preparing for closure towards the end of the project**

☐ Have you communicated when and how involvement will end?

☐ Have contributors been offered opportunities for continued involvement (e.g. dissemination)?

☐ Have you acknowledged and recognised contributions (e.g. feedback, outputs, certificates)?

**After the project**

☐ Will contributors receive updates on outputs and impacts?

☐ Have you created or considered an alumni-style network or mailing list?

☐ Have you signposted future opportunities for involvement?

☐ Have you been transparent about what can and cannot be sustained without funding?
